# Three-dimensional evaluation of the effects of injectable platelet rich fibrin (i-PRF) on alveolar bone and root length during orthodontic treatment: a randomized split mouth trial

**DOI:** 10.1186/s12903-021-01456-9

**Published:** 2021-03-02

**Authors:** Talar S. Zeitounlouian, Kinan G. Zeno, Bassel A. Brad, Rania A. Haddad

**Affiliations:** 1 Department of Orthodontics and Dentofacial Orthopedics, Faculty of Dental Medicine, Damascus University, Damascus, Syria; 2 Division of Orthodontics and Dentofacial Orthopedics, American University of Beirut Medical Center, P.O. Box 11-0236, Riad El-Solh, Beirut, 1107 2020 Lebanon; 3 Department of Oral and Maxillofacial Surgery, Faculty of Dental Medicine, Damascus University, Damascus, Syria

**Keywords:** Injectable platelet rich fibrin, i-PRF, Platelet concentrate, Class II division I, Maxillary canine retraction, Alveolar bone preservation, Root resorption, Dehiscence, Fenestration

## Abstract

**Background:**

The role of injectable platelet rich fibrin (i-PRF) in orthodontic treatment has not been investigated with focus on its effect on dental and bony periodontal elements.

**Objective:**

To evaluate the efficacy of i-PRF in bone preservation and prevention of root resorption.

**Methods:**

A randomized split-mouth controlled trial included 21 patients aged 16–28 years (20.85 ± 3.85), who were treated for Class II malocclusion with the extraction of the maxillary first premolars. Right and left sides were randomly allocated to either experimental treated with i-PRF or control sides. After the leveling and alignment phase, the canines were retracted with 150gm forces. The i-PRF was prepared from the blood of each patient following a precise protocol, then injected immediately before canine retraction on the buccal and palatal aspects of the extraction sites. Localized maxillary cone beam computed tomography scans were taken before and after canine retraction to measure alveolar bone height and thickness and canine root length (indicative of root resorption), and the presence of dehiscence and fenestration. Paired sample t-tests and Wilcoxon signed rank tests were used to compare the changes between groups.

**Results:**

No statistically significant differences in bone height, bone thickness were found between sides and between pre- and post-retraction period. However, root length was reduced post retraction but did not differ between sides. In both groups, postoperative dehiscence was observed buccally and palatally and fenestrations were recorded on only the buccal aspect.

**Conclusions:**

I-PRF did not affect bone quality during canine retraction or prevent canine root resorption. I-PRF did not reduce the prevalence of dehiscence and fenestration.

*Trial registration* ClinicalTrials.gov (identifier number: NCT 03399760. 16/01/2018).

## Introduction

Orthodontists have committed various technologies to accelerate tooth movement for the twin purpose of avoiding the side effects of protracted intervention and meeting the patients’ aspiration for shorter treatment [1]. Tooth movement is essentially a biological response to a physical stimulus; speeding up this response should avoid the occurrence of common iatrogenic effects such as white spot lesions, caries, root resorption and periodontal problems [2–5]. Adequate alveolar bone volume and root length are prerequisite conditions for successful orthodontic tooth movement (OTM) and post treatment stability [6].

Surgical approaches to achieve faster OTM, particularly decortication, have been effective [7–10] yet unpopular or not indicated because of their invasiveness and side effects such as post-operative pain, discomfort, swelling and loss of periodontal tooth support [11]. Biomaterials like platelet rich plasma (PRP) and fibrin (PRF) have been advocated as promising alternatives to accelerate OTM with less risk of bone and periodontal loss [12–16] because of their high contents of growth factors that play an important role in angiogenesis, wound healing and bone regeneration [17].

PRF is the second generation of platelet concentrates and has the advantage of gradual release of growth factors that last for up to 28 days [17]. The injectable i-PRF is the liquid form of the substance that is obtained through low-speed centrifugation and has many advantages over the conventional form such as higher rates of regenerative cells and growth factors [18]. The potential benefits of PRF have been widely investigated in dentistry (e.g. bone regeneration and grafting) [19] but remain controversial in the orthodontic field [20, 21].

The side effects of orthodontic treatment on alveolar bone and root length have been extensively studied in the past utilizing two-dimensional methods, which have known limitations such as distortion and over or under estimation [6, 22, 23]. More accurate evaluations of bone level and root resorption can be achieved qualitatively and quantitatively through cone-beam computed tomography CBCT [24–27]. Accordingly, similar 3D radiography is the optimal method to study OTM with the application of biomaterials that may enhance the bone regeneration process and reduce the incidence of gingival invagination and alveolar bone resorption, side effects that may be worsened following tooth extractions, because of reduced blood supply in the extraction site [20].

The effects of i-PRF on the periodontium during orthodontic treatment have not been investigated. We hypothesized that the reported benefits of i-PRF might have an impact on the stability of the bone because of the inherent ability to produce biological mediators differently from other biomaterials, and because of the direct mode of delivery. Accordingly, our aim was to evaluate the efficacy of i-PRF on alveolar bone dimensions, root length (depicting root resorption), as well as bone dehiscence and fenestration, following canine retraction in the sites of extracted first premolars.

## Materials and methods

A randomized single center clinical trial was conducted after obtaining the approval by the institutional review board and ethical review committee of Damascus University (IRB NO.2473). All interventions and data collection methods in this study were performed following the institutional review board guidelines. The study consisted of a split mouth design with (1:1) allocation ratio and was conducted at the Department of Orthodontics clinics in the Faculty of Dentistry. The Consolidated Standards of Reporting Trials (CONSORT) statement was followed for this investigation, which was registered at Clinicaltrials.gov (ID# NCT03399760. 16/01/2018).

Power analysis was performed to calculate the sample size necessary to detect significant differences with a power of 90% at a permissible (α) error of 5% using G*Power 3.1.3 software (Heinrich-Heine-Universitӓt, Düsseldorf, Germany). Sample size calculation was performed based on previous studies [28, 29]. Accordingly, 21 subjects were needed to participate in the study. Written informed consents for participation were obtained from the patients or their parents or legal guardians for those under 18 years of age upon their agreement to take part in the study. In Addition, written informed consent for publishing of X-rays and dental records were also obtained from the participants or their parents.

The inclusion criteria were: post pubertal and adult patients who had Class II division I malocclusion treated with the extraction of the maxillary first premolars; no missing teeth except third molars; no previous orthodontic treatment; absence of systemic diseases and dentofacial anomalies; normal platelet count; optimal oral hygiene (plaque and gingival index ≤ 1). Excluded were patients with the following conditions: taking anticoagulants or medication that interferes with orthodontic tooth movement (NSAIDs); smoking; pregnancy; presence of bony defect radiographically; any restorations or endodontic treatments on maxillary canines; the presence of artifacts on the CBCT scans that impeded the visualization of dental structures.

**Fig. 1:**
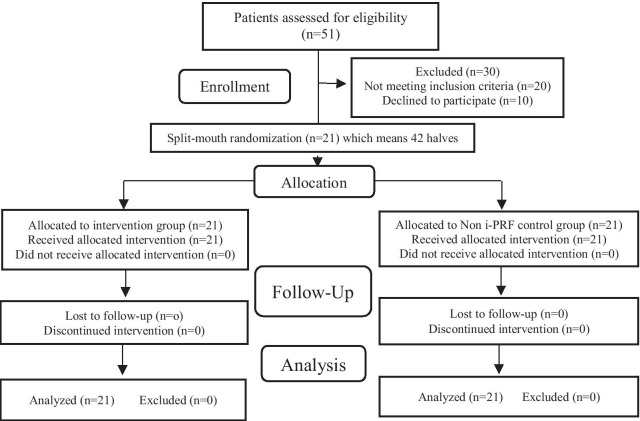
CONSORT flow diagram

Fifty-one patients were screened, and the study objectives were explained to them; 10 patients refused to participate and 20 did not meet the inclusion criteria (Fig. 1). Therefore, a total of 21 patients were recruited, 15 females and 6 males, whose ages ranged between 16 and 28 years (mean 20.85 ± 3.85 years). Upon signing the informed consent, and on the basis of computer aided randomization, the experimental (PRF-injected) or control sides were allocated in the participating patient by an independent coordinator who was not involved in the study.

### Orthodontic procedures

Full mouth scaling was performed in the first visit and instructions given to maintain optimal oral hygiene. Leveling and alignment were achieved with the following archwire sequence in 0.022-in slot size with MBT prescription (Votion, Ortho-Technology, West Columbia, SC, USA): 0.014-in NiTi or 0.016-in NiTi depending on the amount of crowding, 0.016 * 0.022-in NiTi, 0.017 * 0.025-in NiTi, 0.019*0.025-in SS. Progression to next archwire followed the assessment that the previous size was no longer active. A soldered trans-palatal arch was used to maintain the transverse dimension.

The maxillary first premolars were extracted before inserting the 0.019 * 0.025-in SS archwire. Then canine retraction was initiated on both sides before the healing of the sockets 2 weeks after the extraction (T0) to allow the wire to be more passive, using NiTi closing coil springs with a force of 150 g [30]. The total study spanned a period of ten months (April 2018-February 2019), accounting for the initial leveling and alignment phase. Accordingly, the canine retraction duration was recorded between T0 and when the canines were entirely retracted (T1), at which time new dental casts were taken to evaluate the distal movement of the canines following a standardized method. The retraction distance differed between patients, but not between sides, because of varying spaces between the canines and the second premolars across the malocclusions. Tooth displacement was measured on standardized photographs of the maxillary arch using the AudaxCeph software (version 3.4.2.2710; Orthodontic software suite, Ljubljana, Slovenia) following the method of Ziegler and Ingervall [31]. The antero-posterior movements of the canines and molars were recorded from the canine cusp tips and first molar central fossae to a plane intersecting the medial end of third palatal rugae, which have been shown to provide a relatively stable reference [32]. A direct measurement from canine to molar would have confounded the canine distal movement with the molar mesial displacement.

**Fig. 2:**
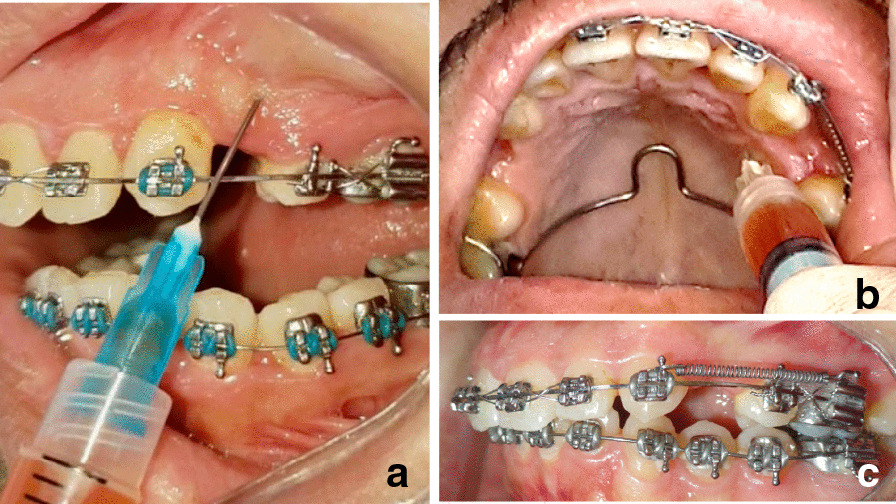
Intraoral photograph of i-PRF injection technique: **a** buccal injection, **b** Palatal injection, **c** Canine retraction initiated using a NiTi coil activated after the first i-PRF injection

Before the initiation of canine retraction (T0), i-PRF was obtained from 20 ml of blood drawn from each patient’s brachial vein, centrifuged at 700 rpm for 3 min [19] and injected on the buccal and palatal aspects of the experimental extraction sites simulating the method of infiltrative local anesthesia injections. The i-PRF injections, equidistant from the maxillary canine and second premolar, were repeated after one month (Fig. 2).

### CBCT imaging and measurements

Maxillary CBCT scans were taken at T0 and T1 utilizing VATECH® (Pax-i3D Green. Seoul, Korea). All scans were captured under constant settings and were of 0.2 mm voxel size. Patients were positioned with their Frankfort plane parallel to the floor. Limited field of view scans were taken that only included the area of interest to minimize exposure to unnecessary radiation. In addition, the pretreatment orthodontic radiographs of the participants were limited to a panoramic and lateral cephalogram excluding the routine posteroanterior cephalogram and periapical radiographs.

**Fig. 3:**
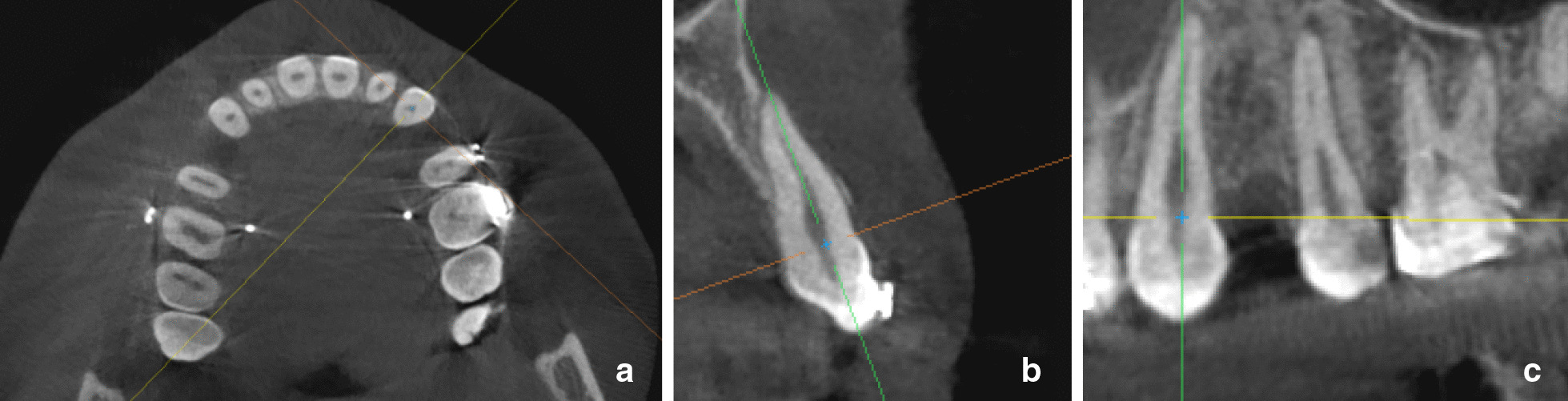
Three-dimensional orientation of the CBCT scans on different sections relative to the position of the upper canine. A: Axial section: B: Coronal section:, C: Sagittal section

One investigator (TSZ) performed all scan measurements blinded to the patient's name and time point by deidentifying the scans. Images were viewed and measured with the software Ez3Dplus (Seoul, Korea) in multi-planar reconstruction mode that contained four different views: axial, coronal, sagittal, and 3D. To obtain comparable and reproducible measurements, all scans were systematically oriented on the different sections (Fig. 3). In the axial plane the intersection between the x and y axes was held in the center of the buccolingual axis of the oval section of the canine that bisected the pulp chamber. In the coronal plane the x-axis passed through the buccolingual CEJ of the canine and the y-axis was parallel to the long axis of the root. In the sagittal plane the x-axis passed through the mesiodistal cementoenamel junction (CEJ) of the canine and the y-axis was parallel to the long axis of the root.

**Fig. 4:**
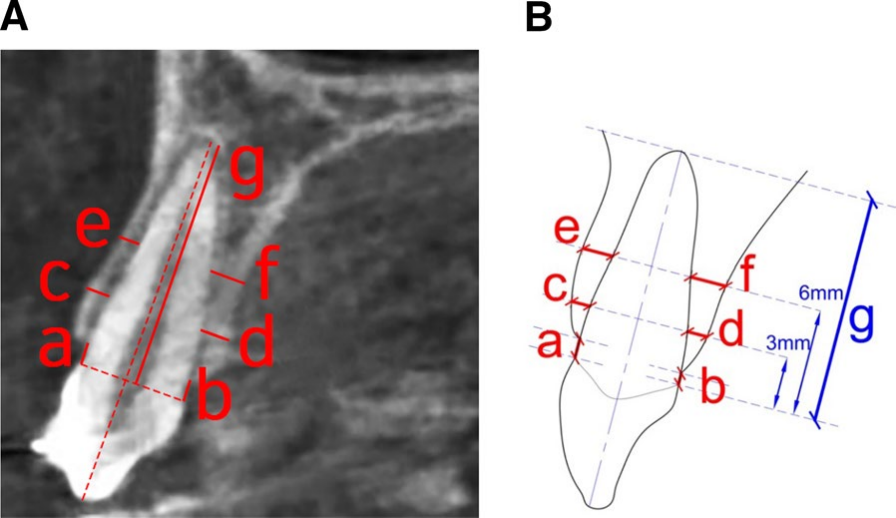
**A** Bucco-palatal section of canine CBCT scan with the defined landmarks for measurements in red. **B** Schematic representation of the reported measurements. Alveolar bone height: buccal (a) and palatal (b) alveolar crest heights were measured as the vertical distances between the line passing through the buccal and palatal CEJ to the corresponding buccal and palatal alveolar crest. Alveolar bone thickness: buccal and palatal alveolar plate thicknesses were measured perpendicular to the long axis from the root surface to the corresponding buccal and palatal alveolar bone plate at two different distances from the CEJ: 3 mm (c, d) and 6 mm (e, f). Root length was measured as the distance (g) along the long axis of the tooth perpendicular to two reference lines, the first line passing through the buccal and palatal CEJ of the canine and the other line through the canine root apex

Measurements at T0 and T1 were taken on the same sections following previously published methods [24, 26, 33, 34]. The primary outcome measures were the alveolar bone crest levels and thicknesses. Buccal and palatal alveolar bone crest level loss (BACL and PACL) was measured as the vertical distance from the buccal and palatal CEJ of the canine to the buccal and palatal alveolar bone crest (the most coronal point of the alveolar bone) parallel to the long axis of the tooth [26] (Fig. 4a, b). The buccal and palatal bone plate thicknesses (BBPT and PBPT) were measured perpendicularly to the long axis of the canine at two different levels: 3 and 6 mm apical to the CEJ [34] (Fig. 4c–f).

The secondary outcomes were canine root length as indicative of resorption, and the incidence of the bony dehiscence and fenestration. Root resorption (RR) was measured by the perpendicular distance along the long axis of the tooth between two reference lines, one passing through the buccal and palatal CEJ and the other through the canine root apex [24] (Fig. 4g). Dehiscence was defined as an increase in the distance from CEJ to the alveolar crest by more than 2 mm; fenestration was reported as a bone defect that exposed areas of the root in at least three consecutive views without involving the alveolar crest [33]. To evaluate intra-examiner reliability, 20 scans were randomly selected and remeasured after one month of the first measurement by the same assessor (TSZ).

### Statistical analysis and error of the method

The intraclass correlation coefficient was computed for intra-examiner reliability assessment of repeated measurements, and the paired t-test was used to calculate systematic and random errors of the latter. The Shapiro–Wilk normality test was applied, and the data were compared with a paired t-test when normally distributed, and with the Wilcoxon Signed when not normally distributed. Statistical analysis was performed using the IBM SPSS Statistics version 25 (SPSS Inc. Chicago, IL, USA); probability values equal or less than 0.05 were considered significant.

## Results

The intraclass correlation coefficients were ≥ 0.937 for all measurements, revealing high reliability. Also, no statistically significant differences were found between the repeated measurements for any variable. The duration of canine retraction was (3.28 ± 1.00) months on the experimental side and (3.57 ± 1.16) months on the control side; the difference between both was not statistically significant.

The T0–T1 difference in movement of the canine was not statistically significant (*P* = 0.918) between the experimental (3.90 ± 1.36 mm) and control (3.94 ± 1.12 mm) sides. Likewise, the T0–T1 differences in buccal and palatal CEJ-alveolar bone crest distances were not statistically significant between experimental and control sides nor between treatment changes (*P* > 0.05), although the reduction was greater on the experimental side and on the palatal aspects (Table 1). Differences in bone thickness between experimental and control sides at both levels measured apical to the CEJ (3 and 6 mm) also were not statistically significant between T0 and T1 (Table 2).

**Table 1:** Means and SD of buccal (BACL) and palatal (PACL) alveolar bone crest level

Variables	N	T0	T1	T0–T1	t value	*P* value^†^
*Experimental side*						
BACL	21	1.71 ± 0.93	1.80 ± 0.96	− 0.09 ± 0.44	− 0.98	0.33
PACL	21	1.66 ± 1.12	1.91 ± 1.14	− 0.25 ± 0.89	− 1.32	0.20
*Control side*						
BACL	21	1.58 ± 0.97	1.62 ± 1.10	− 0.03 ± 0.44	− 0.39	0.70
PACL	21	1.90 ± 1.28	2.02 ± 1.11	− 0.12 ± 0.52	− 1.08	0.29
*Difference between sides*						
BACL	42			− 0.05 ± 0.64	− 0.40	0.69
PACL	42			− 0.13 ± 0.91	− 0.66	0.51

*SD* standard deviation

^†^Paired t test

^*^Significant *P* < 0.05

**Table 2:** Means and SD of buccal (BBPT) and palatal (PBPT) bone plate thicknesses at levels 3 mm (3) and 6 mm (6) above CEJ

Variables	N	T0	T1	T0–T1	z value	*P* value^†^
*Experimental side*						
BBPT (3)	21	0.93 ± 0.30	1.02 ± 0.53	− 0.09 ± 0.37	− 1.21	0.22
BBPT (6)	21	0.86 ± 0.40	0.98 ± 0.62	− 0.11 ± 0.33	− 1.30	0.19
PBPT (3)	21	1.01 ± 0.39	1.00 ± 0.38	0.01 ± 0.34	− 0.28	0.77
PBPT (6)	21	2.16 ± 0.71	2.05 ± 0.67	0.10 ± 0.48	− 0.56	0.57
*Control side*						
BBPT (3)	21	0.96 ± 0.43	0.95 ± 0.57	0.009 ± 0.27	− 1.09	0.27
BBPT (6)	21	0.93 ± 0.53	0.95 ± 0.69	− 0.014 ± 0.34	− 0.57	0.56
PBPT (3)	21	0.79 ± 0.43	0.70 ± 0.37	0.08 ± 0.40	− 0.65	0.51
PBPT (6)	21	1.87 ± 0.69	1.80 ± 0.59	0.06 ± 0.45	− 0.82	0.40
*Difference between sides*						
BBPT (3)	42			0.009 ± 0.27	− 1.52	0.12
BBPT (6)	42			− 0.014 ± 0.34	− 0.92	0.35
PBPT (3)	42			0.08 ± 0.40	− 0.72	0.47
PBPT (6)	42			0.06 ± 0.45	− 0.09	0.92

*SD* standard deviation

^†^Wilcoxon signed rank matched pairs test

^*^Significant *P* < 0.05

The lengths of the retracted canines within each side were statistically significantly reduced at T1 compared to T0 (*P* < 0.001, Table 3). However, differences were minor and not statistically significant different between sides. Dehiscence was more prevalent in both groups postoperatively on the buccal and palatal aspects, while fenestrations were observed on only the buccal aspect on both experimental and control sides (Table 4).

**Table 3:** Means and SD of the canine root length (mm) between experimental and control sides

	N	T0	T1	T0–T1	T value	*P* value^†^
Experimental side	21	15.99 ± 2.07	15.47 ± 2.05	0.51 ± 0.48	1.90	0.000*
Control side	21	16.18 ± 2.14	15.40 ± 2.45	0.78 ± 0.93	3.81	0.001*
Difference between sides				− 0.26 ± 1.04	− 1.14	0.266

*SD* standard deviation

^†^Paired t test

^*^Significant *P* < 0.05

**Table 4:** Incidence of dehiscence and fenestration at the canine level on experimental and control sides

Variable	Intervention side, n (%)			Control side, n (%)		
	T0	T1	T0–T1	T0	T1	T0–T1
*Dehiscence*						
Buccal	5 (23.80)	9 (42.85)	4 (19.05)	4 (19.04)	6 (28.57)	2 (9.53)
Palatal	2 (9.5)	4 (19.04)	2 (9.54)	3 (14.28)	5 (23.80)	2 (9.52)
*Fenestration*						
Buccal	9 (42.85)	10 (47.61)	1 (4.76)	7 (33.33)	7 (33.33)	0 (0)
Palatal	0 (0)	0 (0)	0 (0)	0 (0)	0 (0)	0 (0)

## Discussion

Negative orthodontic side effects on periodontal health have been documented in many studies employing CBCT [35]. This study compliments this body of knowledge in a different perspective, the utilization of biomaterials in an attempt to minimize the negative effects associated with orthodontic treatment while possibly accelerating tooth movement. I-PRF has high contents of growth factors that enhance tissue healing and regeneration such as transforming growth factor β (TGF-β) and platelet-derived growth factor (PDGF) that showed a direct correlation with platelet amount, whereas vascular endothelial growth factor (VEGF) as well as fibroblast growth factor-2 (FGF-2) have a low correlation with platelet count [36]. In addition type 1 collagen gene expression, platelets and lymphocytes are increased [37].

Findings on PRF accelerated orthodontics are controversial. Tehranchi et al. [15] and Nemtoi al. [14] revealed an increased rate of canine retraction when placing L-PRF clot in the extraction sites. Upon moving the maxillary canines in the premolars extraction sites that were preserved with L-PRF membranes, Pacheco et al. [38] found a decreased rate of retraction in 15 out of 17 patients (88%); only two subjects had greater retraction on the experimental side. The conflicting results may be related to different centrifugation protocols and methodology.

Our results do not support the premise of accelerated tooth movement or reduction of side effects with the injection of i-PRF under the present research conditions. This outcome should be viewed in the perspective of the inability to date to promote tooth movement through enhancement of the biological process. Such an intervention represents a prime intellectual and translational logical process, considering that regardless of the physical stimulus applied to affect tooth movement, the entire response is biological within the surrounding periodontal tissues [1]. The balance of current evidence favors the surgical approach (e.g. decortication) because it breaks the physical resistance offered mainly by the compact bone [39]. The implication of the regional acceleratory phenomenon (RAP) in speeding tooth movement may not be totally correct, as RAP might provide mainly the healing process of the decorticated bone [40]. Many variables must be explored to promote acceleration, including the combination of reducing the physical barrier and the application (through injection or other ways) of already or not yet investigated biological substances.

More specifically, alveolar crest height was reduced on both experimental and control sides by less than 1 mm (Table 1), demonstrating that i-PRF did not preserve alveolar bone height. It remains unknown whether different results might be produced by varied rates of injection of this substance (more than at T0 and one month later), such as more rhythmic deliveries throughout the retraction process, the injection of the substance rather placing it in a clot in the extraction site in direct contact with the bone or combining it with a bone graft in a static situation that would warrant its stability and efficiency. The injectable form of PRF produces the highest numbers of platelets and leukocytes compared to the other types [18], yet possibly not rich enough in leukocytes to affect growth factors and cytokines release from the platelet concentrates and subsequently alter the regenerative process significantly. A novel horizontal centrifugation protocol could produce greater amounts of platelets and leukocytes than the fixed angle centrifugation method that we used to generate the PRF and which might cause more damage to cellular contents during centrifugation [41]. These variables warrant further study.

A comprehensive comparison of present findings with those in the literature should consider studies with and without the use of biomaterials. In most studies without the introduction of biomolecules, orthodontic treatment following tooth extraction was associated with loss of alveolar bone height and morphologic dimensional changes that could not be avoided [25, 33, 42]. However, Baxter et al. [43] did not observe differences between the extraction and non-extraction treatments. Among only a few publications of orthodontic treatment combined with the application of biomaterials to enhance the alveolar bone, Liou [44] showed in case reports of en-masse retraction of anterior teeth and mandibular molar protraction that the injection of PRP submucosally decreased alveolar bone loss. Using CBCT measurements, Alomari and Sultan [45] found that PRP did not reduce alveolar bone loss on the buccal surfaces of the maxillary first premolars and molars after rapid palatal expansion. In a CBCT study to gauge bone quality following orthodontic treatment without enhancement with biomaterials, Lund et al. [25] observed significant buccal and palatal (lingual) alveolar bone loss following premolar extractions on all teeth except the maxillary canines, which displayed a loss of only 0.1 mm on the buccal side and 0.6 mm on the palatal side. These findings are in agreement with ours, although the authors reported pre- and post-orthodontic treatment measurements whereas ours were before and after canine retraction. In contrast, our results also indicated that the alveolar bone maintained its original thickness. In untreated patients, Lee et al. [46] found the mean CBCT-measured buccal and palatal alveolar bone thicknesses of the maxillary canines to be greater than 1 mm buccally and palatally at the 3 mm level apical to the CEJ, nearly double our findings. This difference may be attributed to different malocclusions or ethnicity (Korean in Lee et al.’s study; Caucasian in this investigation).

The reduction of canine root length by less than 1 mm on both experimental and control sides is likely related to the fact that space closure is accompanied with increased apex movement and the susceptibility to root resorption in malocclusions treated with extraction when compared with non-extraction treatment [23–26, 47]. In a similar CBCT study of maxillary canine root resorption, Abdel Kader et al. [29] found a statistically significant reduction of 0.54 ± 0.77 mm, which may not be of clinical significance. Our results are also in agreement with the results of Jiang et al. [48] who studied the canine root resorption through volumetric CBCT following retraction in the extracted premolar space utilizing sectional mechanics (T Loop), and with the findings by Harris and Baker [23] who reported in the treatment of Class II, division 1 with premolar extraction a mean maxillary canine root length reduction of less than 1 mm on two dimensional radiographs.

The increased dehiscence on the buccal experimental side (42.85%) and less on the palatal side (19.04%) are similar in trend (although not in amount) with Castro et al.’s rates of 60% and 5%, respectively [26]. Evangelista et al. [33] supported the findings that maxillary canines and first premolars were the most susceptible teeth to dehiscence and fenestrations, also reporting greater buccal bony defects when compared with the palatal side. Hyo-Won et al. [49] found during en-masse retraction of the anterior teeth a higher prevalence of dehiscence on the palatal side than on the labial side of the maxillary incisors (67–68% palatal and 2–14% labial), and the canines (32% palatal and 12% labial). The greater prevalence than in our study on the buccal side might be related to the retraction method (en-masse versus single tooth retraction).

Bone density was evaluated on CBCT images in a study in which platelet rich fibrin plugs were placed in the maxillary first premolar extraction socket [14]. The authors reported a faster movement by only 1 mm over a period of 6 months compared to the contralateral control side, and the prevalence of more homogeneous cortical bone with increased density (> 1250 Hounsfield units) on the experimental side two months post-extraction. This result contradicts the finding that an increase in bone density takes longer to become obvious radiographically [50]. Nevertheless, it may indicate better preservation of the socket, warranting another level of investigation in this field.

The forces used in this study were within the acceptable limits prescribed by other authors [16, 30]. CBCT has been used in major studies to evaluate more precise tooth movement three-dimensionally because it is a very accurate, reliable and sensitive technique to evaluate bone changes [33]. Indeed, the data in those reports and in this study could not be obtained with precision with two-dimensional radiographs. Protective measures were taken to reduce exposure to radiation, including limited field of view scans and reduction of the number of regular x-rays routinely taken prior to treatment, measures deemed acceptable by the IRB and explained to the patients or their parents or legal guardians for those under 18 years of age prior to their informed consent.

A few limitations are observed in this study. Gender related differences and patient-centered outcomes such as levels of pain and discomfort were not addressed, requiring further investigation. Longer follow up periods are required to evaluate the long-term effect of repeated (at various times) PRF injections on the integrity of alveolar bone and root resorption, as well as its therapeutic capacities for alveolar bone remodeling. Although within the frame of the power analysis to detect significant differences, the relatively small sample size might have masked significant outcomes. Further studies are needed to better elucidate all possible effects of platelet concentrate and related products on various orthodontic procedures and comparing the effects of different types of application and centrifugation protocols on alveolar bone.

## Conclusion

Within the conditions and limitations of this study, the following conclusions are drawn:

- Injectable PRF is not an effective method to maintain the alveolar bone dimensions or to prevent root resorption in orthodontic treatment involving tooth extraction.
- Injectable PRF did not reduce the levels of dehiscence and fenestrations.
- Acceleration of tooth movement through local biological substances has not yet been proven successful for clinical application and warrants various research approaches.

## Data Availability

The datasets used and/or analyzed during the current study are available from the corresponding author on reasonable request.

## Abbreviations

3D: 3 Dimensional
BACL: Buccal alveolar bone crest level
BBPT: Buccal bone plate thicknesses
CBCT: Cone-beam computed tomography
CEJ: Cemento-enamel junction
CONSORT: Consolidated Standards of Reporting Trials
i-PRF: Injectable platelet rich fibrin
IRB: Institutional review board
NSAID: Non-steroidal anti-inflammatory drugs
OTM: Orthodontic tooth movement
PACL: Palatal alveolar bone crest level
PBPT: Palatal bone plate thicknesses
PDL: Periodontal ligament
PRP: Platelet rich plasma
RAP: Regional acceleratory phenomenon
RR: Root resorption
